# Bimanual coordination during a physically coupled task in unilateral spastic cerebral palsy children

**DOI:** 10.1186/s12984-018-0454-z

**Published:** 2019-01-03

**Authors:** Sharah A. Mutalib, Michael Mace, Etienne Burdet

**Affiliations:** Department of Bioengineering, Imperial College London, South Kensington, London, SW7 2AZ UK

**Keywords:** Bimanual coordination, Physical coupling, Unilateral spastic, Cerebral palsy, Rehabilitation

## Abstract

**Background:**

Single object bimanual manipulation, or physically-coupled bimanual tasks, are ubiquitous in daily lives. However, the predominant focus of previous studies has been on uncoupled bimanual actions, where the two hands act independently to manipulate two disconnected objects. In this paper, we explore interlimb coordination among children with unilateral spastic cerebral palsy (USCP), by investigating upper limb motor control during a single object bimanual lifting task.

**Methods:**

15 children with USCP and 17 typically developing (TD) children performed a simple single-object bimanual lifting task. The object was an instrumented cube that can record the contact force on each of its faces alongside estimating its trajectory during a prescribed two-handed lifting motion. The subject’s performance was measured in terms of the duration of individual phases, linearity and monotonicity of the grasp-to-load force synergy, interlimb force asymmetry, and movement smoothness.

**Results:**

Similar to their TD counterparts, USCP subjects were able to produce a linear grasp-to-load force synergy. However, they demonstrated difficulties in producing monotonic forces and generating smooth movements. No impairment of anticipatory control was observed within the USCP subjects. However, our analysis showed that the USCP subjects shifted the weight of the cube onto their more-abled side, potentially to minimise the load on the impaired side, which suggests a developed strategy of compensating for inter-limb asymmetries, such as muscle strength.

**Conclusion:**

Bimanual interaction with a single mutual object has the potential to facilitate anticipation and sequencing of force control in USCP children unlike previous studies which showed deficits during uncoupled bimanual actions. We suggest that this difference could be partly due to the provision of adequate cutaneous and kinaesthetic information gathered from the dynamic exchange of forces between the two hands, mediated through the physical coupling.

## Background

*Bimanual coordination* is a task-specific and active assembling procedure where two hands are restricted to act cooperatively by virtue of mutual coupling [1]. For typically developing (TD) children, the process of acquiring bimanual skills develops through environmental exploration and object manipulation during activities of daily living (ADL or ADLs), such as learning to unwrap a piece of candy or tying shoelaces [2]. Unfortunately, for children with unilateral spastic cerebral palsy (USCP), the resulting motor impairments after a paediatric brain injury significantly reduce their ability to learn and adapt bimanual actions into ADLs like their TD counterparts [3]. These limitations are predominantly attributed to muscle weakness, spasticity and sensorimotor impairment to one side of their body, caused by damage to the contralateral side of the motor cortex, a part of the brain that maintains the coordination, precision and timing of a movement [4, 5]. Furthermore, more than 95% of children with USCP display poor sensory function in their upper limbs, such as reduced proprioception, tactile discrimination and stereognosis [6, 7], which often disrupt their anticipatory and sequencing of grasp force control [8]. As a result, children with USCP require more time, planning and concentration to perform bimanual manipulation activities compared to TD children [9].

Bimanual training has been proven effective for children with USCP, as from a neurological perspective, it potentially can encourage interhemispheric communication and improve ipsilateral motor cortex activation to the affected hemisphere [10, 11]. Bimanual manipulation tasks can be carried out whilst handling either a single object or multiple objects at the same time. A single object bimanual task, defined as a *physically coupled bimanual task*, involves co-manipulation of a mutual object, such as squeezing a rubber ball with both hands. According to [12], in order for a task to be considered (physically) coupled, the movement from one limb must have an effect on the dynamics of the opposite limb. Therefore, such a task provides additional sensorimotor information whereby each individual hand can sense the force generated by the other hand through coordinated interaction with the object. In contrast, a multiple (normally two) object task, defined as an *uncoupled bimanual task*, involves each hand moving independently to manipulate two (or more) separate objects. Unlike physically-coupled tasks, there is no direct transfer of the forces between the hands, hence no cuteneous and kinaesthetic feedback of the opposing hand’s actions.

The predominant research focus has been on tasks which encourage the two hands to act independently by manipulating separate objects and on the analysis of the motor control mechanisms during these uncoupled bimanual tasks. Such studies demonstrated that a congruent task, where both hands perform the same activity (either in phase or in anti-phase), improved the grasp force modulation and timing of the impaired limb relative to an equivalent unimanual task [13, 14]. On the other hand, an incongruent task, where the two hands act separately to perform different actions, caused a significant deterioration of the control of grasp force on the less-impaired side [15]. The current consensus is that the less-impaired side usually performs within the normal TD range during both unimanual and congruent bimanual tasks [8, 16].

Although arguably more realistic, physically-coupled bimanual task, where the hands are haptically connected through a single object, has never previously been explored in USCP populations. In everyday activities, bimanual tasks are often performed with the hands physically coupled due to the size, weight or function of the object. Examples include opening a screw-top jar or lifting a large box. Furthermore, performing physically-coupled bimanual tasks provide additional sensorimotor information compared to uncoupled bimanual tasks. For instance, [17] showed that redundant information obtained by both hands could improve haptic estimates. This suggests that manipulating an object with two hands could off-load processing of cutaneous and kinaesthetic information to the ipsilateral side, through coordinated force transfer between the hands and the object. The same forces would not be transferred between the limbs when exploring two independent objects alone. This ‘ipsilateral strengthening’ of sensorimotor activation could help children with USCP compensate for their impaired sensory function, hence improving anticipation and sequencing of force control [8]. Moreover, some children with USCP are biased towards their less-impaired side during ADLs, whilst the impaired side are often neglected - a phenomenon known as developmental disregard [18]. Therefore, physically enforcing both hands to work together in a natural way could provide a simple method of ‘unlocking’ potential functionality of the impaired limb.

Haptic feedback is vital for the spatial and temporal coordination between the two limbs regardless of age and/or impairment [19]. Studies on upper limb coordination during physically coupled bimanual tasks with hemiparetic stroke patients found that the individuals improved the overall smoothness, speed and accuracy of their movements [20, 21]. [21] discovered that there is a potential benefit from the proprioceptive component provided by haptically coupling both hands to assist movement. However, it is unknown whether physically coupled tasks could yield similar benefits in children with USCP and is, therefore, the focus of the present study.

In the present study we examined temporal, force and kinematic coordination between the two hands in USCP affected and TD children, during a physically coupled lifting task. Of particular interest is the analysis of how children with USCP would carry out a lifting task when the two hands are physically and therefore haptically coupled. We hypothesised that children with USCP would potentially benefit from haptic information relayed between the two hands, to compensate for their impaired sensory function. Children with USCP were also anticipated to develop a specialisation strategy based on the superior capabilities of the less-impaired hand. Considering the sensorimotor impairment due to USCP, we also hypothesised that these children would have impaired temporal coordination when initiating and terminating subsequent movement phases, reduced force modulation, and lower movement smoothness than TD children. The temporal, force and kinematic characteristics of the lifting movements were measured using a bespoke instrumented cube.

## Methods

***Participants***

A total of 32 children participated in this experiment. The *experimental group* consists of 15 (9M/6F) children with cerebral palsy (mean age 8.7 y, standard deviation 2.7 y, range 4.8 - 13.5 y; see Table 1 for participant information) diagnosed as unilateral spastic according to the Surveillance of Cerebral Palsy in Europe (SCPE)’s classification [22]. The *control group* consisted of 17 (7M/10F) typically-developing (TD) children (mean age 8.2 y, standard deviation 2.5 y; range 4.4 - 13.8 y) with no known medical disorders or history of neurological or musculoskeletal problems. During recruitment, subjects were selected to ensure that the means and standard deviations of the two groups were similar.

**Table 1:** Descriptive information for the USCP subjects

Participant	Sex	Age (y)	Hemiparetic side	Spasticity (wrist)	MACS level	Tactile sensibility
1	F	9.5	Left	0	1	T
2	F	10.9	Right	1	1	D
3	F	4.8	Right	1+	2	I
4	F	13.5	Left	1+	2	D
5	M	9.1	Right	2	3	D
6	M	11.0	Left	2	3	I
7	F	5.5	Right	1+	2	I
8	F	9.3	Left	1	1	T
9	M	10.1	Left	2	1	D
10	M	5.0	Right	1	1	T
11	M	11.1	Left	1+	2	D
12	M	8.9	Left	1	3	I
13	M	11.2	Left	2	3	D
14	M	5.9	Left	1+	2	I
15	M	5.5	Right	1	2	I

M, male; F, female; T, typical; D, decreased; I, impaired

Subjects with USCP were chosen based on their ability to grasp and lift an object with both hands and their ability to follow instructions. Spasticity level of subjects with USCP was assessed at the wrist using the Modified Ashworth Scale ranging from 0 to 4 [23]. To test the mirror movement of subjects with USCP, a short unimanual reaching and grasping test was conducted on the less-impaired hand, whilst observing the mirror-symmetric activity on the impaired hand. None of the children exhibited overt mirror movements during unimanual movements. The tactile sensibility of all subjects was measured using a two-point discrimination test and the results were interpreted as 3 mm = typical, 7 mm = decreased, and > 7 mm = impaired [24]. All TD children had typical tactile sensibility.

All subjects gave verbal assent and informed consent was obtained from the parents before their participation in the experiment. The experiment was performed in accordance with the ethical standards laid down in the 1964 Declaration of Helsinki and was approved by the Imperial College Research Ethics Committee and the University of Sultan Zainal Abidin Research Ethics Committee.

***Measurement tool***

**Fig. 1:**
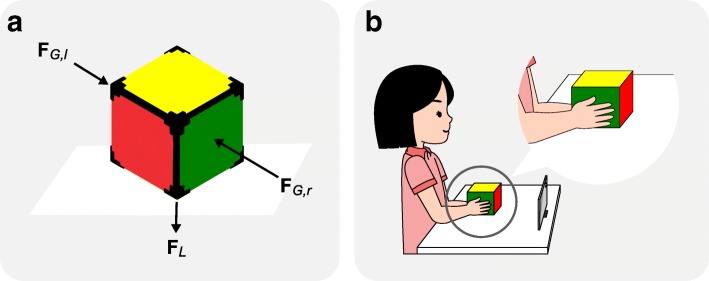
The measurement device and the sitting position of the subject. Shown are **a** the measurement device and the estimated locations of grasp and load forces applied on the cube, and **b** the sitting posture and the estimated hand positions of the subjects

The measurement tool used in this experiment is a bespoke instrumented cube of dimension 10 cm × 10 cm × 10 cm and weight 530 g (Fig. 1a). The cube’s size has been selected to make it impossible for the subjects to lift it with one hand alone. Each face of the cube was equipped with a force transducer (TAL107F, HT Sensor Technology Co., Ltd, linear range 0-98 N, hysteresis ± 0.05%), to measure the force applied on each side of the cube. Additionally, a 9-degree of freedom Bosch BNO055 Inertial Measurement Units (IMU) was placed inside the cube to estimate its absolute orientation and linear translation. Data from the force sensors and the IMU were recorded wirelessly over Bluetooth at 60 Hz and 10-bit resolution. A custom data collection program running on an Android tablet was created in the Unity game engine (Unity Technologies, USA).

***Experimental procedure***

The experiments were conducted individually in a quiet room with minimal distractions. Each subject was seated comfortably on a chair in front of a table where the cube was placed. The chair was adjusted in height so as to have the forearms horizontal when the cube was grasped. Figure 1b shows the sitting posture of the subject and the position of the hands relative to the shape of the cube. Prior to the data recording, the force signals from the cube were zeroed when on the table, to remove the spurious offsets and the interaction between the bottom force sensor and the table. Each subject was asked to squeeze the cube as hard as he/she can, in order to record the maximum grasp strength. Subjects were then instructed to grasp the cube with both hands, move it vertically for approximately 8-10 cm, hold for 1–2 s, and then return back to the start position. The movements were demonstrated prior to data collection. A total of 15 self-paced trials were recorded from each child, with a 10-second interval between trials. The first five trials for each subject were considered practice trials and excluded from the data analysis.

***Data analysis***

As visualised in Fig. 1a, grasp force *F*_*G*_ was measured from the average left and right forces on the cube (F _*G*,*l*_+ F _*G*,*r*_)/2, and load force (tangential force) *F*_*L*_ was measured from the interaction between the bottom face of the cube and the table. All signals from the left, right and bottom force sensors, as well as the linear acceleration signal from the IMU were filtered using a 4th order low-pass Butterworth filter with a 10 Hz cut-off.

**Fig. 2:**
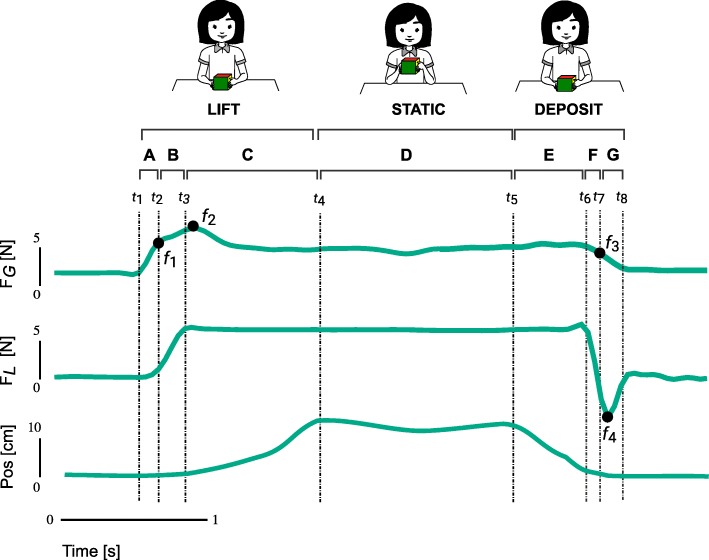
Segmentation of data into seven task phases. Shown are the grasp force *F*_*G*_, load force *F*_*L*_ and position (pos) signals plotted as a function of time, from one TD subject aged 10.1 y. The signals were divided into 7 phases; (A) preload, (B) load, (C) ascend, (D) static, (E) descend, (F) unload and (G) postload. The phases between A to D are collectively identified as ‘lift’, and E to F as ‘deposit’. The descriptions of the time (*t*) and force (*f*) events are presented in Table 2

Figure 2 shows the segmentation of *F*_*G*_ and *F*_*L*_ signals from one TD subject aged 10.1 y. Data from all trials were divided into 7 phases; (A) preload, (B) load, (C) ascend, (D) static, (E) descend, (F) unload, and (G) postload. The phases between A to D are collectively identified as ‘lift’, and E to F as ‘deposit’. The preload phase started at the onset of *F*_*G*_> 0.2 N, *t*_1_, and continued until the onset of cube’s lift off, *t*_2_, marked by *F*_*L*_> 0.2 N. During the load phase, *F*_*G*_ and *F*_*L*_ increased isometrically until the cube is fully lifted, *t*_3_, during which *F*_*L*_ reads the weight of the cube, 5.1 N. The cube then began to ascend, remain static for 2 s and descend. The unload phase began when the bottom face of the cube touches the table again at *t*_6_, and *F*_*L*_ signal reads < 5.1 N. *F*_*G*_, together with *F*_*L*_, continued to decrease isometrically throughout the unload phase. The cube was fully deposited on the table at the onset of postload phase, *t*_7_, where *F*_*L*_< reads 0.2 N. The phase ended when both hands were fully released from the cube at *t*_8_, where *F*_*G*_ signal reads <0.2 N.

**Table 2:** Description of temporal and force parameters

Parameters	Description
Time Parameters	
*t*_1_	Onset of grasp force >0.2*N*
*t*_2_	Onset of loading, load force >0.2*N*
*t*_3_	Time when the cube is fully lifted off the table
*t*_4_	Onset of static phase, cube stops moving upwards
*t*_5_	Onset of unloading, cube starts moving downwards
*t*_6_	Time when cube first touched the table
*t*_7_	Cube fully unloaded on the table
*t*_8_	Grasp force fully released
Force Parameters	
*f*_1_	Grasp force at the onset of positive load force (N)
*f*_2_	Peak grasp force (N)
*f*_3_	Grasp at the onset of cube’s contact with of table (N)
*f*_4_	Reversed force during unloading (N)

The duration of each phase and the magnitude of the force parameters were recorded and analysed. The descriptions of each timing and force parameters are described in Table 2. Further analyses were performed based on the force and kinematic characteristics. All data analysis were performed in MATLAB^®^ (The MathWorks, USA).

***Motor control measures***

**Isometric grasp force-load force synergy** The relationship between the isometric grasp force and load force, during the load *t*∈*B* and unload *t*∈*F* phases were computed from an ordinary least square regression model for each subject and trial, with grasp force *F*_*G*_ as the dependent variable and load force *F*_*L*_ as the predictor variable in the form

1 $$ F_{G} = \alpha_{0} + \alpha_{1} F_{L} +\varepsilon   $$

where *α*_0_ is the intercept, *α*_1_ the linear slope, and *ε* the unexplained variance of the load force.

The synergy between grasp force and load force were analysed in term of *linearity* and *monotonicity*. The linearity of the grasp force-load force synergy *γ*_*GL*_, which measures the error from the grasp-load relationship, was calculated from the mean absolute error of Eq. 1, in the form

2 $$ \gamma_{\text{GL}} = - \frac{1}{N}\sum_{k=1}^{N} \left| F_{G}[k] - \hat{F}_{G}[k] \right|,  $$

where *N* is the total number of observations and is different for loading and unloading time periods. *F*_*G*_[*k*] and $\hat {F}_{G}[k]$ are the grasp force and predicted linear force at time *k*-th, respectively.

The monotonicity of the grasp force-load force synergy *μ*_*GL*_, in the direction of the principal axis *p*, which measures how smoothly the grasp-load forces evolve together, was calculated as

3 $$ {}\begin{aligned} \mu_{GL} &= \frac{1}{N} \sum_{k=1}^{N-1} dI[k] \\ dI[k] &= \left\{\begin{array}{ll} 1 & \text{if}\,\, \bar{F}_{G}[k+1] - \bar{F}_{G}[k] >0 \\ -1 & otherwise \end{array}\right. \end{aligned}  $$

where $\bar {F}_{G}$ is the rotation of *F*_*G*_ matrix based on the angle *θ*, with

4 $$ {}\begin{aligned} \left[\begin{array}{c} \bar{F}_{G}\\ \bar{F}_{L} \end{array}\right] &= R(\theta) \cdot \left[\begin{array}{c} F_{G} \\ F_{L} \end{array}\right], \quad R(\theta) = \left[\begin{array}{cc} \cos\theta & -\sin\theta \\ \sin\theta & \cos\theta \end{array}\right],\\ \theta &= \tan^{-1} \left(\alpha^{1}\right) \end{aligned}  $$

where the angle *θ* is calculated from the gradient from the *α*_1_ in Eq. 1.

**Inter-limb force asymmetry** The symmetry of forces produced by the preferred hand and non-preferred hand during lift *t*∈[*A*:*C*] and deposit *t*∈[*E*:*G*] were analysed using a linear mixed-effects model. The bias in grasp force between the preferred and the non-preferred hand *F*_*B*_ were the dependent variable and the total force from the preferred and non-preferred hands *F*_*T*_ were the predictor, in the form

5 $$ F_{B} = \beta_{0,i} + \beta_{1,i} F_{T} +\varepsilon_{i}   $$

where *β*_0,*i*_ is the intercept, *β*_1,*i*_ the linear slope, and *ε*_*i*_ the unexplained variance of the total force, which vary independently for each subject *i*. For each subject, *F*_*B*_ and *F*_*T*_ were normalised to the maximum grasp force produced. The overall interlimb force asymmetry for USCP group and TD group were compared by adding the group factor *G* in the model,

6 $$ F_{B} = \beta_{0,i} + \beta_{1,i} F_{T} + \beta_{2,i} F_{T} G + \beta_{3,i} G + \varepsilon_{i}   $$

which was assumed to affect the linear slope and the intercept of the force symmetry curve. For TD children, the dominant hand was deemed as the preferred hand. For children with USCP, the less-impaired hand was deemed as preferred hand while the impaired as the non-preferred hand.

**Smoothness of movement** The Spectral Arc Length (SPARC) metric [25] was computed on the angular velocity data during ascend phase *t*∈*C* and descend phase *t*∈*D*, to determine the smoothness of the respective movements.

***Statistical analysis***

Due to the relatively small sample size and the non-Gaussian distribution of the variables of interest, the median value was thus chosen as a representative measure to ensure the robustness of our statistical tests. Non-parametric statistical tests were used, such as a Mann-Whitney *U* test, to determine the significant differences between the USCP and TD groups, and a Wilcoxon signed-rank test, to determine statistical differences between the movement phases for both USCP and TD groups. For the analysis of interlimb asymmetry, a likelihood ratio test was computed to determine the difference between Eqs. (5) and (6). Significance was calculated at the 5% level and all data for the time, force, and kinematics coordination analysis are reported as median ± interquartile range (USCP vs TD).

## Results

***Force coordination and timing***

**Fig. 3:**
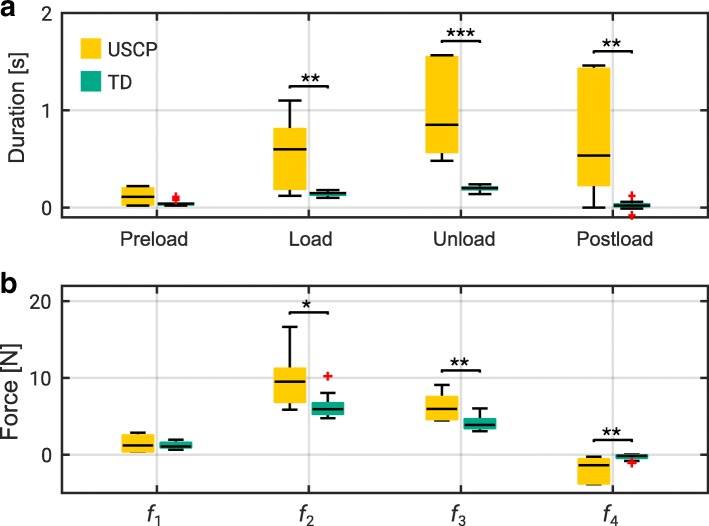
Time and force parameters for TD group and USCP group. Shown are **a** the duration of each task phase and **b** the magnitude of each force parameter, as defined in Fig. 2

Figure 3 shows the median ± interquartile range of (a) the duration of each phase and (b) the force magnitude parameters of children in USCP and TD groups. No significant difference in the duration of preload phase was observed between USCP and TD children (0.11 ± 0.19 s vs 0.04 ± 0.02 s; USCP vs TD; *p* = 0.16). The time taken by children with USCP during the load phase was approximately four times longer than TD children (0.60 ± 0.64 s vs 0.15 ± 0.04 s; *p* < 0.001). The latency is even longer during unload (0.85 ± 1.00 s vs 0.20 ± 0.05; *p* < 0.001) and postload (0.54 ± 1.22 s vs 0.02 ± 0.05 s; *p* < 0.01).

A Mann-Whitney U test revealed insignificant difference to the amount of grasp force at the onset of cube’s lift off, *f*_1_ between children in USCP and TD group (*p* = 0.86). However, children with USCP generated greater grasp force during lifting, *f*_2_ (9.52 ± 4.35 N vs 5.93 ± 1.40 N; *p* < 0.05) and greater grasp force at the onset of unloading, *f*_3_ (5.97 ± 2.91 N vs 3.89 ± 1.17 N; *p* < 0.01). The negative load force, *f*_4_, which suggests the cube being pressed against the table during unloading, is greater among children with USCP than TD children (-1.38 ± 3.18 N vs -0.16 ± 0.34 N; *p* < 0.01).

***The linearity and monotonicity of grasp force-load force synergies***

**Fig. 4:**
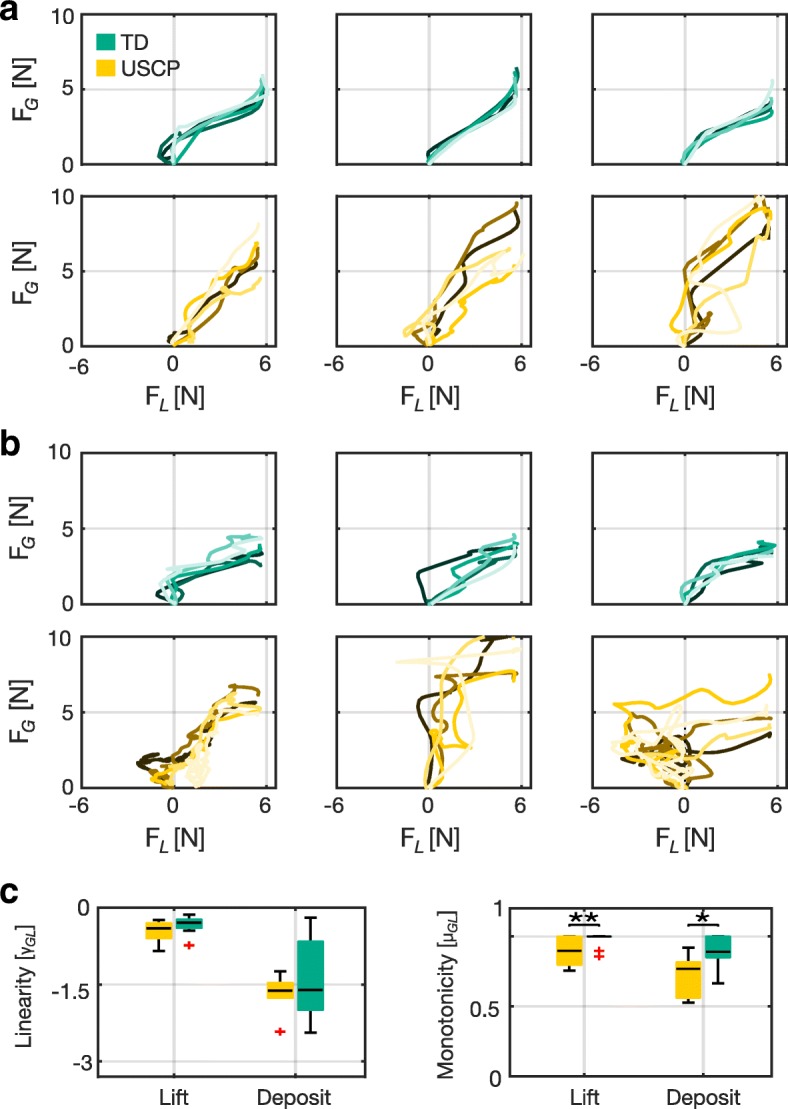
Grasp force-load force synergies of TD and USCP subjects during the physically coupled lifting task. Shown are the grasp force signals plotted against the load force signals during **a** preload + load phases, *t*∈[*A*:*B*] and **b** unload + postload phases, *t*∈[*F*:*G*]. Five trials were superimprosed for three TD and three USCP subjects. **c** Shows the linearity and monotonicity scores of TD and USCP subjects during load and unload phases

Figure 4 shows the isometric grasp force signal plotted against the load force signal during (a) preload and load phases, and (b) unload and postload phases. TD children demonstrated consistent increases in grasp and load forces during both the load and unload phases, with only small variations in isometric force development between subsequent lifts. The loading phase is preceded by a short preload phase, during which the grasp force increased, sometimes in conjunction with a small negative load force, when the cube was pressed down against the surface.

Figure 4c shows the median and interquartile range of the linearity and monotonicity scores during load phase and unload phase. The linearity scores for children in USCP group remain indifferent from TD children during both load (0.40 ± 0.29 vs -0.29 ± 0.15; *p* = 0.2) and unload (-1.72 ± 0.28 vs -1.80 ± 1.32; *p* = 0.92). However, the monotonicity scores of children with USCP were lower than TD children during both load and unload (0.90 ± 0.20 vs 1 ± 0; *p* < 0.01 and 0.77 ± 0.25 vs 0.89 ± 0.15; *p* <0.05, respectively). A Wilcoxon signed-rank test revealed no significant improvement in the linearity and monotonicity scores of the grasp force-load force synergies between the first and the last trials in both TD and USCP groups.

***Interlimb force asymmetry***

**Fig. 5:**
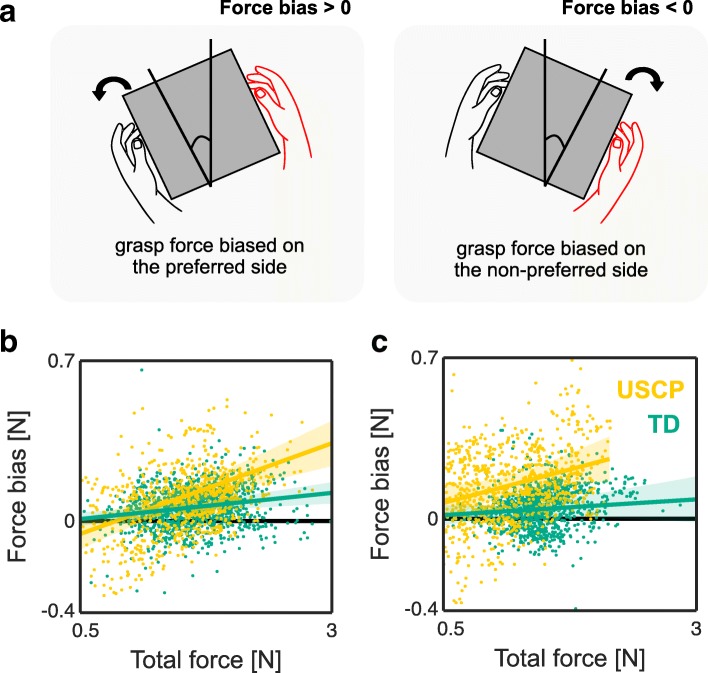
The analysis of interlimb force asymmetry. Shown is **a** the illustration of the effect of cube’s rotation on the force bias magnitude. **b** and **c** show the plot of force bias as a function of total force during lift phase *t*∈[*A*:*C*] and deposit phase *t*∈[*E*:*G*], respectively. The shaded region represents 95% confidence interval of the linear mixed-effects model fit

Figure 5b and c show the result from the linear mixed effect model of the force bias between the preferred and the non-preferred hand as a function of the total force from both hands during the lift and deposit phases, respectively. Overall, the children with USCP and TD children showed bias towards the preferred hand during lift and deposit. A linear mixed-effect analysis revealed significant effect of USCP on the interlimb force asymmetry curve compared to TD children during lift (*χ*^2^(2) = 5.71; *p* = 0.04). Children in USCP group exhibit lower intercept by 0.06 ± 0.04 N and higher linear slope by 0.06 ± 0.03 relative to TD group. The lower intercept by children with USCP at the beginning of the lift highlights the hand that initiates the grasping, the preferred hand, causing the cube to fall on the non-preferred hand within the first few seconds of the lift. Similarly, there was a significant effect from impairment on the interlimb force asymmetry curve during the deposit phase (*χ*^2^(2) = 14.80; *p* < 0.001), in which children in USCP group increased the linear slope by 0.09 ± 0.05 relative to that produced by the TD group.

***Smoothness of movement***

**Fig. 6:**
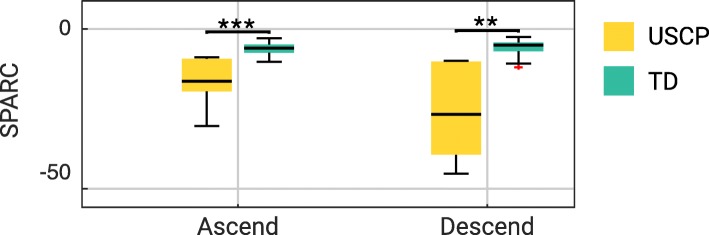
The SPARC scores of TD and USCP subjects. Shown are the SPARC scores during ascend and descend phases. Higher SPARC scores represent smoother movements

Figure 6 shows the SPARC score for both TD and USCP children during (a) object ascend, and (b) descend. Children in USCP group exhibit lower SPARC scores than TD group during ascending and descending movements (-16.37 ± 0.24 vs -5.99 ± 2.66; *p* < 0.001 and -26.75 ± 37.55 vs -5.08 ± 2.78; *p* < 0.01, respectively).

## Discussion

Previous studies have discussed the efficacy of bimanual tasks in improving upper limb coordination of children with USCP, as the less-impaired side could act as a ‘template’ to assist the movement of the impaired side [26]. For instance, a number of studies reported improved anticipation and sequencing of force control of the impaired side during a bimanual task, compared to an equivalent unimanual task [13, 14], although others noted that this improvement occurred at a temporal cost, where the less-impaired hand slowed down to match the impaired hand [14]. However, these studies only focused on the coordination of the upper limbs during a bimanual task that involves manipulating separate objects on each hand. In this paper, we aimed to further explore the potential of bimanual tasks on the interlimb coordination of children with USCP, by analysing the performance of both hands during a physically coupled task, where both hands are constrained to interact with a single object.

The quantification of the grasp force-load force synergy is a simple but powerful method to analyse the adaptation of grasp forces to environmental demands [27]. Such demands include the weight and size of an object, and the friction between the object’s surface and skin. In order to produce smooth and dexterous prehension, the development of isometric grasp and load forces must be scaled to match the object’s weight [28]. TD children normally use a strategy which harmonises the isometric grasp and load forces by automatically adjusting to the object properties, producing a coordinated or linear grasp-load force output [29]. Conversely, children with CP usually exhibit a significantly more non-linear synergy, compared to TD children, during a unimanual grip task, which can be attributed to multiple factors, such as inefficient sensorimotor integration and deficits in anticipatory control [11, 29]. However, our results could not endorse the findings from these studies, as we found that the linearity of the grasp force-load force relationship by USCP subjects falls within the normal TD range, potentially suggesting that the USCP subjects could generate grasp force in a coordinated manner when performing our physically-coupled task.

Our findings may be explained by the fundamental principles that govern grasp force control. Proper control of grasp force relies on intact sensorimotor integration, as it provides the nervous system with information about the object’s physical properties [27]. This information allows for anticipatory scaling of the grasp force level to adjust subsequent prehension. However, sensory function is frequently disrupted in USCP children [6], thus affecting their anticipatory control. To compensate, children with USCP rely on external sensory feedback for grasping, which means the grasp force will increase excessively to amplify the sensory feedback prior to the object’s lift-off (i.e. *f*_1_) [29]. Potentially, this excess of grasp force could excite additional tactile mechanoreceptors by increasing the skin-object contact surface. Such compensation strategies often lead to prolonged preload duration to permit preparation for the lifting phase [29]. However, our study found neither excessive *f*_1_ grasp force magnitude nor prolonged preload duration during lifting in the USCP group, suggesting no evidence of impaired anticipatory control. Together, the absence of signs of impaired anticipatory control and the linear grasp-load force synergy could be an indication of adequate sensory information obtained during the task, suggesting that the physical coupling of the hands during the manipulation of a single object could facilitate natural cutaneous and kinaesthetic information relayed between the impaired and healthy limbs.

The monotonicity of grasp force-load force synergy is a metric introduced in this study to provide further analysis of the grasp-load force synergy. In contrast to linearity, which measures the performance error from the overall grasp-load relationship, the measure of monotonicity elucidates the efficiency of voluntary control of isometric muscle contractions when initiating and terminating a movement. A monotonic grasp-load force relationship involves a gradual contraction and relaxation of muscle with precise coordination of agonistic and antagonistic muscles. We have shown that children with USCP have difficulty generating isometric grasp force patterns in a monotonic manner, indicating frequent grasp - ungrasp oscillations during each load or unload phase. It has been established before that muscle weakness is one of the major problems that present in many CP cases [30] - and the oscillating grasp force patterns discovered within children with USCP could possibly be a result of increased muscle noise that occurs due to decreased muscle strength [31]. Potentially, the weak muscle prominent in USCP children could have contributed to the decrease in overall smoothness of the movement itself, as we discovered that these children produced a jerky and unsteady movement during object lift and deposit. However, the results from the analysis of interlimb symmetry show the strategy taken by children with USCP to compensate for their weak muscle, where an asymmetry distribution of grasp force during lift and deposit phases suggests that these children shifted some of the cube’s weight onto the more-abled side, in order to reduce the load on the impaired side.

However, further work should be conducted to elucidate the specific role that haptic information plays during physically coupled bimanual tasks and its effect on the anticipatory control of children with USCP. This could be done by modulating the strength of physical connection (i.e. using a robotic interface) between the two hands whilst performing the same task, and investigating how different coupling strengths affect an individual’s control. In addition, a previous study on healthy adults has shown how reaching for a visually unified object (rather than two separate objects) has decreased planning time and enhanced anticipatory control [32]. Unfortunately, the visual factor was not considered in this study. A prototypical bimanual task that eliminates the influence of visual feedback should, therefore, be designed to validate the role of haptic feedback in improving the anticipation of an object’s physical properties and the sensorimotor control of grasp force.

## Conclusion

This study provides new evidence towards the understanding of impaired bimanual function among children with USCP. It has been previously highlighted that executing bimanual tasks will activate the intact brain hemisphere, enabling neural crosstalk at different levels of the central nervous system [1, 33]. Our findings show that by physically coupling the hands during a symmetric bimanual task, normal anticipation and sequencing of grasp force control, can be observed in USCP affected subjects which suggesting further ipsilateral strengthening of the intact hemisphere. We hypothesize that this is facilitated by the dynamic exchange of forces between the two hands through the manipulation of a single mutual object by reflecting natural cutaneous and kinaesthetic information from the impaired side. Furthermore, given that most USCP children have ‘grown-accustomed’ to using only one hand during ADLs, by physically enforcing both hands to work together in a natural way, such a task could provide a simple method to increase the functional capacity of the impaired limb.

## Abbreviations

ADLs: Activities of daily living
CP: Cerebral palsy
USCP: Unilateral spastic cerebral palsy
TD: Typically developing
IMU: Inertial measurement unit
SPARC: Spectral arc length
